# Investigating the Origins of Toxic Response in TiO_2_ Nanoparticle-Treated Cells

**DOI:** 10.3390/nano7040083

**Published:** 2017-04-11

**Authors:** Gamze Kuku, Mustafa Culha

**Affiliations:** Department of Genetics and Bioengineering, Yeditepe University, 34755 Atasehir, Istanbul, Turkey; gamzeku@gmail.com

**Keywords:** titanium dioxide nanoparticles, TiO_2_ NPs, nanotoxicity, Surface-enhanced Raman scattering, SERS, cytotoxicity

## Abstract

Titanium dioxide nanoparticles (TiO_2_ NPs) are widely used in sunscreens, cosmetics and body implants, and this raises toxicity concerns. Although a large number of reports claim that they are safe to use, others claim that they induce reactive oxygen species formation and can be carcinogenic. In this study, the origins of toxic response to TiO_2_ NPs were investigated by using Surface-enhanced Raman spectroscopy (SERS) which provides multidimensional information on the cellular dynamics at single cell level without any requirement for cell fixation. Three cell lines of vein (HUVEC), lung carcinoma (A549) and skin (L929) origin were tested for their toxic response upon exposure to 20, 40, 80 and 160 µg/mL anatase-TiO_2_ NPs for 24 h. It was demonstrated that the level of toxic response is both cell line and dose-dependent. L929 fibroblasts were the most resistant cell line to oxidative stress whereas in HUVEC and A549, cell lines collagen and lipid deformation were observed, respectively.

## 1. Introduction

Titanium dioxide nanoparticles (TiO_2_ NPs) have one of the highest production rates among nanomaterials (NMs) annually worldwide and are used in a wide range of applications that expose humans to TiO_2_ NPs from various sources [1]. As cosmetic ingredients, they interact with skin, as food additives and coatings they interact with the gastrointestinal system, and as paint ingredients they can be inhaled through the lungs to reach lymphatic drainage which can then end up in blood circulation.

Among its many application areas, biomedical applications of TiO_2_ NPs account for less than 10% and are mostly used in body implants [1]. TiO_2_ NPs have biocompatibility and strength advantages over other materials in biological environments; they are resistant to body fluid effects that might lead to implant corrosion or rejection whereas they have high tensile strength and flexibility. Although a layer of TiO_2_ NPs naturally occurs on titanium-containing implant surfaces, new studies show that applying a rough surface of TiO_2_ NP coating on the implant surface induces osteoblast adhesion and maturation, which are crucial factors for bone implants [2,3,4]. Moreover, TiO_2_ NPs have been investigated for their applicability in drug delivery, imaging, photodynamic therapy, and biosensor technologies in which promising results were reported [5,6,7,8,9,10].

Apart from their advantageous properties, TiO_2_ NPs have been accepted as “safe” materials and their cytotoxicity is often neglected. In the cases where TiO_2_ NPs were reported to be toxic, they exerted their toxicity in reactive oxygen species (ROS)-dependent pathways such as glutathione (GSH) reduction, increased cell adhesion, changes in carbohydrate metabolism, apoptosis induction and inflammation [11,12,13,14]. In some reports, an induction of autophagy was reported [15]. However, in a study by Lopes et al., it was also shown that the downstream steps of autophagy was actually blocked when human keratinocyte cell line (HaCaT) was incubated with TiO_2_ NPs and autophagosomes accumulated within the cells [16]. Other reports demonstrate that genotoxicity might be induced by TiO_2_ NPs [17,18]. A short survey on the toxicity of TiO_2_ NPs in the literature might lead to confusion due to conflicting reports. Some show that TiO_2_ NPs are toxic whereas others imply that they are safe, with some studies even reporting increased proliferation rates upon exposure [11,19]. A closer look reveals that the origin of these variations can be found in the experimental details that were also summarized by Golbamaki et al. as the variations in; (i) the size; (ii) size distributions; (iii) purity levels and (iv) surface areas of NMs with same average sizes, as well as the differences in (v) coatings; (vi) crystal structure of the same types of NMs; (vii) aggregate sizes in solution or media; (viii) the assays used for toxicity determination; and (ix) the concentrations of the tested NPs [19].

Taking these points into consideration, more standardized and systematic studies are needed to clearly understand the molecular mechanisms behind the toxic and non-toxic responses that occur upon NP exposure. One approach to learn about these mechanisms is to use surface-enhanced Raman spectroscopy (SERS), a mode of Raman spectroscopy, in which the surface of a nanostructured noble metal is used to enhance Raman scattering [20,21,22]. Since its discovery, SERS was used in a wide range of biological and non-biological applications, demonstrating that it is indeed a powerful technique, especially for biological applications [23,24,25,26,27,28,29,30,31,32,33].

The technique is advantageous for in vitro NP toxicity studies for multiple reasons and has been demonstrated as a promising approach to be further developed [34,35,36]. In addition to the easy sample preparation, being label-free, and having a high spatiotemporal resolution; the technique provides information about the cellular processes in a multidimensional fashion [37,38,39]. This reduces the number of assays to be performed in order to come to a conclusion on the mechanism and extent of the toxic response. It is also possible to target specific intracellular components by modifying the SERS substrates with special targeting ligands such as nuclear localization signal (NLS) peptide and obtain SERS signals from the vicinity of the cell nuclei [40,41].

Therefore, in this study, as an extension of our previous report, we aimed to investigate the possible causes of toxicity upon exposure to increasing concentrations of anatase TiO_2_ NPs through SERS spectral information obtained from three cell lines [34]. The cell lines that were used in the study were human umbilical vein endothelial cells (HUVEC), adenocarcinomic human alveolar basal epithelial cells (A549) and mouse fibroblasts from subcutaneous areolar and adipose tissue (L929) in an attempt to obtain information from cells of vascular, lung and skin origin, respectively.

## 2. Results

### 2.1. Characterization of AuNPs and TiO_2_ NPs

**Figure 1:**
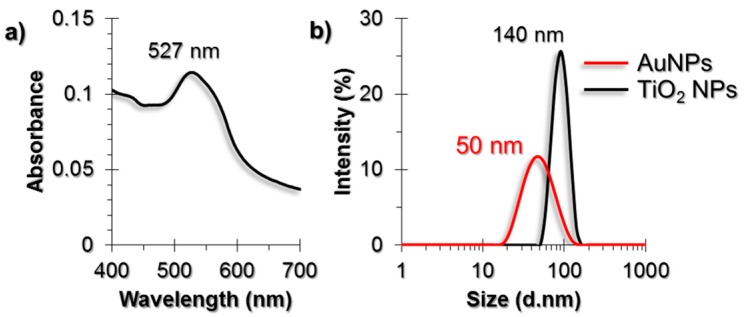

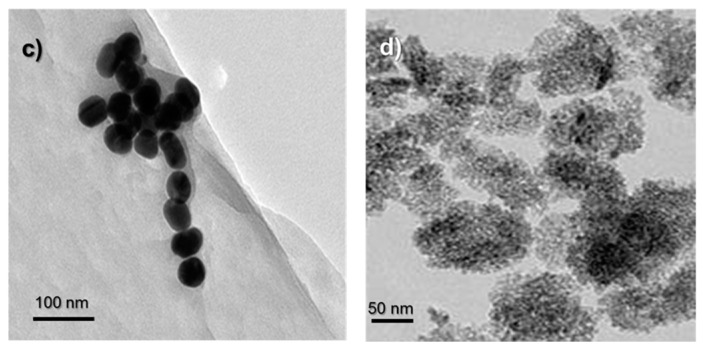
(**a**) UV-Vis spectrum of AuNPs; (**b**) DLS size measurement results of AuNPs and Titanium dioxide nanoparticles (TiO_2_ NPs). TEM images of (**c**) AuNPs and (**d**) TiO_2_ NPs. The scale bars in TEM images are 100 nm for AuNPs and 50 nm for TiO_2_ NPs.

Gold nanoparticles (AuNPs) that were synthesized by the citrate reduction method were characterized by using UV-Visible (UV-Vis) spectroscopy, Dynamic Light Scattering (DLS) and Transmission Electron Microscopy (TEM) techniques (Figure 1). The diameter of AuNPs were found to be around 50 nm. The purchased powder-form TiO_2_ NPs were dispersed in a serum-free cell culture medium by ultrasonication prior to each experiment and were characterized by DLS and TEM. The diameter of TiO_2_ NPs were around 140 nm in DLS measurements.

### 2.2. WST-1 Assay

**Figure 2:**
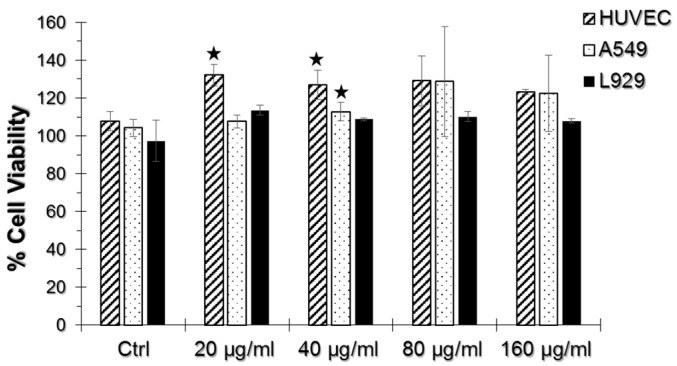
WST-1 assay results of human umbilical vein endothelial cells (HUVEC), A549, and L929 cell lines incubated with 20, 40, 80 and 160 μg/mL TiO_2_ NPs and AuNPs. The % Cell viability of test groups were normalized to their respective non-exposed samples, setting the viability level of non-exposed samples to 100%. Error bars represent ± S.D. and statistically significant groups were marked with a star sign (two-tailed Student’s *t*-test for unpaired samples, *p* ≤ 0.05).

As seen in Figure 2, we did not observe a significant decrease in cell viability upon TiO_2_ NP and AuNP exposure in any of the cell lines tested. There was not any significant viability difference in samples incubated only with TiO_2_ NPs, thus they were not included in the results. Instead, a significant viability increase at 20 and 40 μg/mL TiO_2_ NP and AuNP-incubated HUVECs and 40 μg/mL TiO_2_ NP and AuNP-incubated A549 cells were observed.

### 2.3. SERS Spectra of Cells Incubated with TiO_2_ NPs

**Figure 3:**
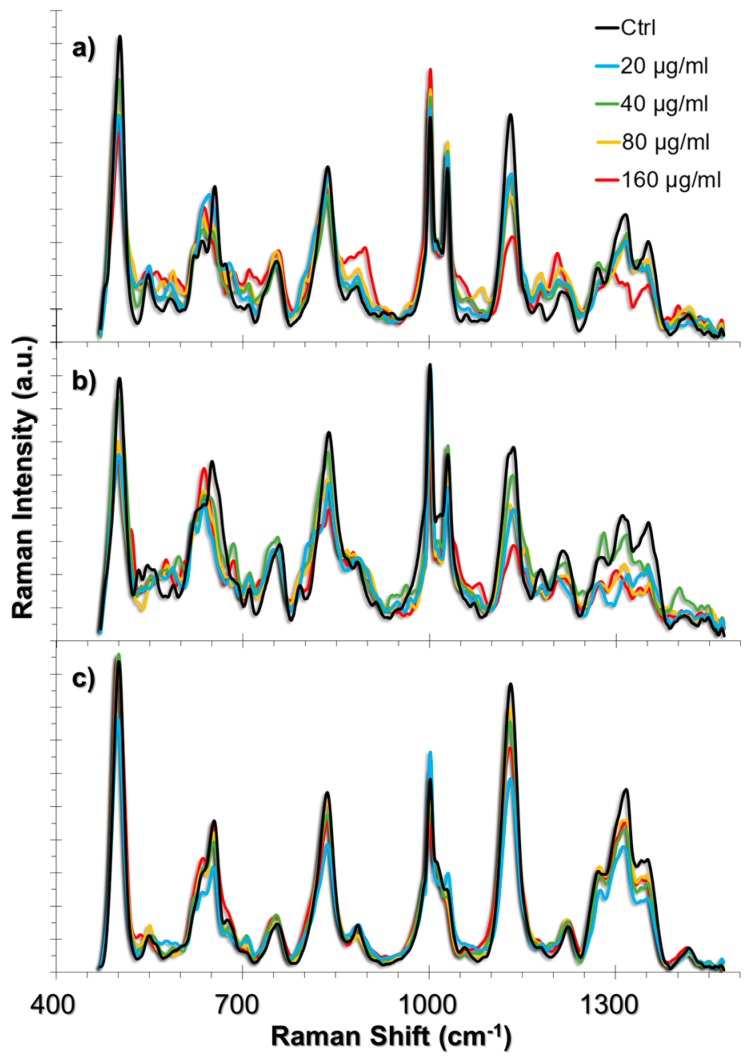
SERS spectra obtained from (**a**) HUVEC, (**b**) A549, and (**c**) L929 cell lines upon 24 h exposure to TiO_2_ NPs. The spectra obtained from increasing concentrations of TiO_2_ NPs were overlapped with their respective non-incubated controls.

The ROS produced in the presence of TiO_2_ NPs interact with the cellular components [42]. Among these components, the cell membrane, extracellular matrix (ECM), and ECM proteins that are expressed in the cell are the first molecular structures that might be affected, whereas antioxidant activity attempts to neutralize these effects [43,44]. In oxidative stress conditions as well as in autophagy, the collagen degradation rate increases [45,46,47]. The degradation takes place in the lysosomes following their uptake through endocytosis. On the other hand, lipids of the cell membrane might be peroxidized and cause membrane deformation. The approach that we used in this study enables the tracking of such events in single living-cells because the AuNPs are also taken up by the cells through endocytosis; we have previously shown that ~50 nm AuNPs were distributed both in the cytoplasm and in the endosomes when they were incubated with the cells for 24 h [34]. Therefore, it is possible to obtain intracellular and endosomal information from the live-cells by establishing the approach provided herein. A summary of the SERS spectra obtained from HUVEC, A549 and L929 cell lines incubated with 20, 40, 80 and 160 g/mL TiO_2_ NPs are given in Figure 3 together with their respective non-incubated controls. The list of peak assignments were provided in Table 1.

#### 2.3.1. SERS Spectral Patterns of HUVEC Cell Line

**Figure 4:**
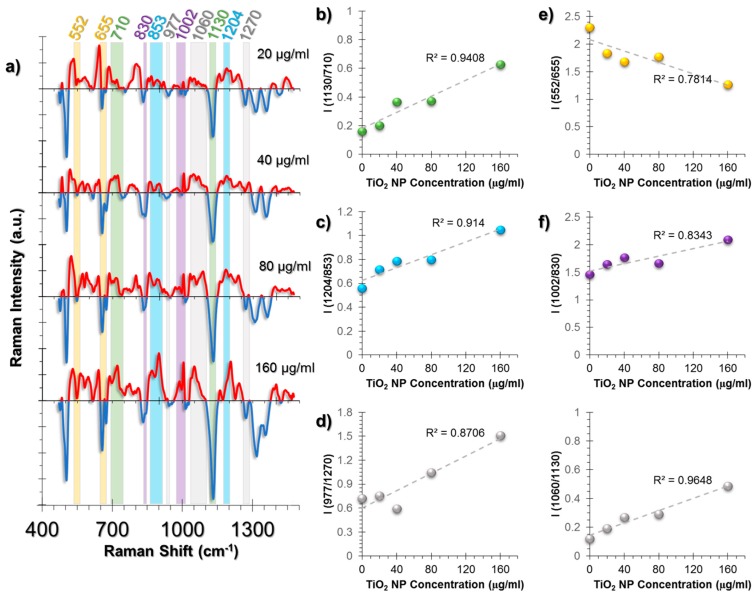
(**a**) The spectra obtained from non-incubated control cells were subtracted from the TiO_2_ NP-incubated cells to allow for easier tracking of spectral changes. The positive values corresponding to a spectral intensity increase were colored red, whereas the negative values were colored blue. Another color code was used to visualize the intensity ratio combinations. The peaks with the same highlight color were compared as an intensity ratio. The intensity ratios obtained from the SERS spectra of HUVEC cell line at increasing concentrations of exposure to TiO_2_ NPs for 24 h. The ratios represent (**b**) membrane changes, (**c**) collagen changes, (**d**) β-sheet to α-helix changes of collagen structures, (**e**) GSH/GSSG antioxidant activity, (**f**) Phe/Tyr inflammation marker, (**g**) triglyceride/phospholipid trans-gauche changes as an inflammation marker. The coefficient of determination (*R*^2^) was given for each graph next to their trendlines.

The major spectral changes in HUVECs were observed at peaks related to lipids, collagen structure, and antioxidant activity of the cells (Figure 4a). The phospholipid trans-gauche isomerization peak (1130 cm^−1^) is an indicator of lipid structural change and decreases with the increased rate of gauche forms; this was observed in our results. A comparison of this peak to the peak at 710 cm^−1^ related to cholesterol and membrane phospholipid head showed a linear and TiO_2_ NP dose-dependent response on the membrane structural changes (Figure 4b).

Collagen is a protein with a triple helix structure and the helices are mostly stabilized by the help of hydroxyproline amino acid residues [49,50]. The changes in this residue result in structural destabilization, leading to collagen deformation and ROS was shown to increase hydroxyproline content [51]. A similar trend was also observed in our study. The intensity ratio of 1204 cm^−1^ hydroxyproline and 853 cm^−1^ proline peaks followed a linear trend (Figure 4c). Moreover, protein β-sheet (977 cm^−1^) to α-helix (1270 cm^−1^) was increased as an indication of collagen deformation (Figure 4d).

The general oxidative stress response in cells is to attempt to neutralize the stress through cellular antioxidants. The major antioxidant in the human body is glutathione (GSH), containing glutamate, a cysteine, and glycine residues [52]. In reduced form, GSH contains a sulfhydryl group from its cysteine residue. However, at oxidizing conditions, it is oxidized to form glutathione disulfide (GSSG). Using this information, the intensity ratio of S-S trans-gauche-trans modes (552 cm^−1^) and C-S stretching (655 cm^−1^) were compared and a linear increase trend was observed, which indicates GSH depletion due to antioxidant activity (Figure 4e). 

Another pattern was observed in the phenylalanine/tyrosine (Phe/Tyr) levels calculated from the ratio 1002/830 cm^−1^ (Figure 4f). Phe/Tyr ratio is an inflammation marker [53]. In the presence of hydroxyl radicals (^•^OH), phenylalanine hydroxylases exert an impaired activity, thus the ratio increases. An additional inflammation marker was also traced from the ratio of Triglyceride/Phospholipid trans-gauche peaks, 1060/1130 cm^−1^ (Figure 4g).

#### 2.3.2. SERS Spectral Patterns of A549 Cell Line

**Figure 5:**
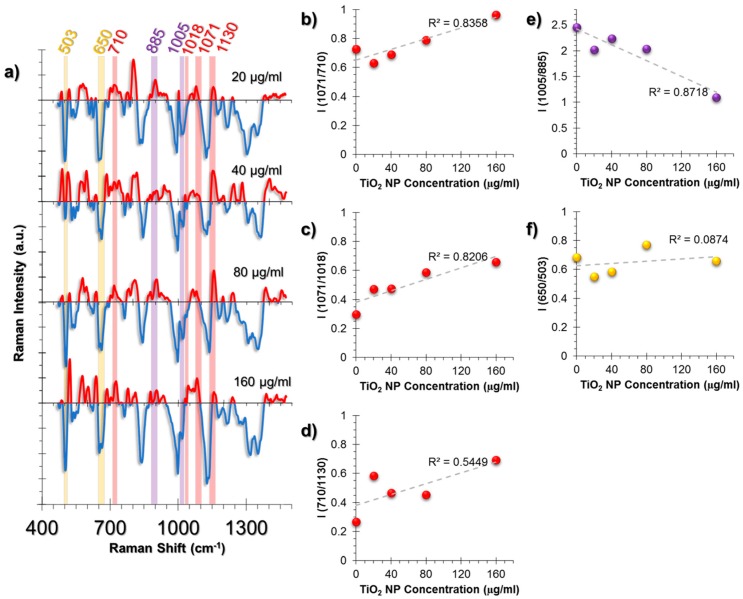
(**a**) The spectra obtained from non-incubated control cells were subtracted from the TiO_2_ NP-incubated cells to provide an easier tracking of spectral changes. The positive values corresponding to a spectral intensity increase were colored red, whereas the negative values were colored blue. Another color code was used to visualize the intensity ratio combinations. The peaks with the same highlight color were compared as an intensity ratio. The intensity ratios obtained from the SERS spectra of A549 cell line at increasing concentrations of exposure to TiO_2_ NPs for 24 h. The ratios represent (**b**) Triglyceride deposition, (**c**) Fatty acid biosynthesis increase, (**d**) Cholesterol/phospholipid trans-gauche membrane changes, (**e**) Phe/Tyr inflammation marker, (**f**) GSH/GSSG antioxidant activity. The coefficient of determination (*R*^2^) was given for each graph next to their trendlines.

The cellular response in the A549 cell line was more lipid-related than HUVECs (Figure 5a). An increase in triglycerides was observed from the intensity ratio of triglyceride (1071 cm^−1^) and cholesterol (710 cm^−1^) peaks (Figure 5b). It is a known phenomenon that in stress conditions cells might deposit triglycerides and that cholesterol levels are decreased compared to triglycerides [54,55]. Moreover, the linear increase of the triglyceride/glycogen ratio (1071/1018 cm^−1^) indicates a switch of metabolic activity to fatty acid biosynthesis due to mitochondrial dysfunction (Figure 5c) [56].

Membrane deformation was calculated from the ratio (710/1130 cm^−1^); the rate of cholesterol (710 cm^−1^) to phospholipid trans-gauche changes (1130 cm^−1^) and the cholesterol levels were found to increase (Figure 5d).

Unlike the HUVEC cell line, the inflammation marker Phe/Tyr levels (1005/885 cm^−1^) were found to decrease (Figure 5e). Moreover, the GSH/GSSG ratio, calculated from 650/503 cm^−1^, was found to remain almost unchanged (Figure 5f).

#### 2.3.3. SERS Spectral Patterns of L929 Cell Line

**Figure 6:**
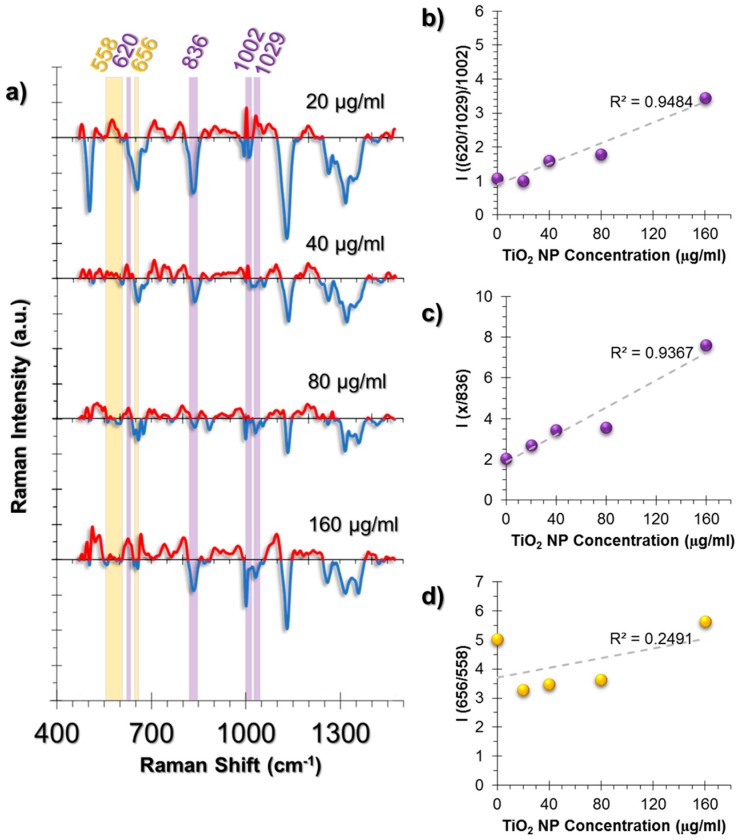
(**a**) The spectra obtained from non-incubated control cells were subtracted from the TiO_2_ NP-incubated cells to provide an easier tracking of spectral changes. The positive values corresponding to a spectral intensity increase were colored red, whereas the negative values were colored blue. Another color code was used to visualize the intensity ratio combinations. The peaks with the same highlight color were compared as an intensity ratio. The intensity ratios obtained from the SERS spectra of L929 cell line at increasing concentrations of exposure to TiO_2_ NPs for 24 h. The ratios represent (**b**) Phenylalanine deformation, (**c**) Phe/Tyr inflammation marker, (**d**) GSH/GSSG antioxidant activity. For the sake of clarity, in graph (c), letter “x” on the *y* axis represents the ratio that was obtained in (**b**), which was named as “Phe deformation”. The coefficient of determination (*R*^2^) was given for each graph next to their trendlines.

The L929 cell line is of fibroblast origin and fibroblasts are known to be more tolerant to oxidative stress than endothelial cells [57,58]. In the obtained SERS spectra, we also observed highly similar spectral patterns among the cells incubated with increasing TiO_2_ NP concentrations (Figure 6a).

On the other hand, two prominent changes were observed. One of them was the deformation of Phe symmetric vibrations calculated from the intensity ratio of C-C aromatic ring twist (620 cm^−1^) and changes in Phe (1029 cm^−1^) to symmetric ring vibrations of Phe (1002 cm^−1^) (Figure 6b). Moreover, the Phe/Tyr ratio, that was calculated from the Phe deformation (denoted as “x” in Figure 6c) divided by 836 cm^−1^ Tyr peak, showed an increasing trend at increasing concentrations of TiO_2_ NPs (Figure 6c).

The second spectral change was observed from the GSH/GSSG ratio calculated from the ratio 656/558 cm^−1^ (Figure 6d). The trend was different from the HUVEC and A549 cell lines. At 20 μg/mL TiO_2_ NP exposure, a sharp intensity ratio decrease was observed compared to the control group. Conversely, the ratio was rescued back to the control group levels at samples incubated with increasing TiO_2_ NP concentrations. This can be interpreted as an indication of the neutralization of the oxidative stress effects. Thus, our observations were in agreement with the literature that states that fibroblasts are less prone to oxidative damage.

## 3. Discussion

Distinct cellular responses were obtained in cell type and dose-dependent manners in this study. Although the WST-1 cell proliferation assay did not reveal a reduction in viability, and it is inconclusive in the TiO_2_ NP toxicity evaluation, the SERS spectra showed that there are actually changes in the cells at the molecular level.

TiO_2_ NPs induce ROS production through Fenton-like reactions which produce a high amount of ^•^OH radicals [59]. The produced radicals then interact with the cellular components and might result in responses such as lipid peroxidation, ECM deformation, apoptosis and inflammation. Therefore, we investigated various peaks for their intensity correlations to enlighten the molecular mechanisms upon TiO_2_ NP exposure. The peak combinations include collagen deformation, antioxidant activity, membrane deformation, Phe/Tyr inflammation marker, protein structural deformation and triglyceride/glycogen oxidative stress ratio.

In light of these comparisons and observations, it can be stated that the L929 cell line was the least affected cell line upon exposure, even though an inflammatory response could be predicted from the Phe/Tyr ratio. However, the antioxidant activity might neutralize the effects and protect the cells from oxidative stress.

Both the HUVEC and A549 cell lines are of endothelial origin even though the prior is from a healthy vein and the latter is from lung carcinoma. Since A549 is a carcinoma cell line, it would be predicted that the cells show a metabolic switch towards fatty acid biosynthesis, store triglycerides and decrease glycogen production, which were observed in the SERS spectra. Moreover, we observed that membrane deformation was more prominent in this cell line compared to HUVECs. The decreased rate of Phe/Tyr, on the other hand, excludes the inflammatory response.

The conversion of proline to hydroxyproline in the presence of ^•^OH radicals is accepted as an oxidative stress-related response of collagen structures. Moreover, antioxidant activity was observed from the reduced GSH/GSSG ratio whereas the increased Phe/Tyr ratio indicated an inflammatory response, as in the case of L929 cells.

In conclusion, although many studies could not detect the toxic response upon 24 h of TiO_2_ NP exposure, by utilizing SERS it was possible to point out the initiation of collagen deformation, inflammation and protein structural deformations in HUVEC, A549 and L929 cell lines. Therefore, we propose SERS as a sensitive tool that can direct researchers to further investigations on nanotoxicity studies which can ultimately reduce the types of experiments that need to be completed.

## 4. Materials and Methods

### 4.1. Cell Culture

Human umbilical vein endothelial cells (HUVEC), adenocarcinomic human alveolar basal epithelial cells (A549) and mouse fibroblasts from subcutaneous areolar and adipose tissue (L929) cell lines were purchased from ATCC. HUVEC cell line was cultured in DMEM-high glucose supplemented with 10% FBS and 1% Penicillin-Streptomycin. A549 and L929 cell lines were cultured in DMEM-F12 supplemented with 10% FBS, 1% Penicillin-Streptomycin and 2 mM l-Glutamine. Cells were incubated in an incubator with 5% CO_2_ humidified atmosphere at 37 °C. All cell lines were checked regularly to ensure that they were mycoplasma-free. For SERS studies, 10,000 cells were seeded on calcium fluoride (CaF_2_) slides which were cut into 1 cm^2^ pieces to prevent fracture of the slides as well as to be able to use 24-well culture plates to place the slides for NP incubation. For WST-1 assay the same amount of cells as for SERS studies were seeded on 96-well plates.

### 4.2. AuNP Synthesis, TiO_2_ NP Dispersion, and Characterization

AuNPs were synthesized following the citrate reduction method [60,61]. To disperse purchased powder form TiO_2_ NPs (Alfa Aesar, catalog no. 45603), serum-free cell culture medium was used; DMEM with high glucose content for HUVEC cells and DMEM-F12 for A549 and L929 cells. Dispersion was carried out for 15 min in a chilled ultrasonicator bath. The dispersed NPs and synthesized AuNPs were characterized using UV-Vis spectroscopy (Lambda 25, Perkin-Elmer, Foster City, CA, USA), DLS (ZetaSizer Nano ZS, Malvern Instruments, Malvern, UK) and TEM (JEM-2100, Peabody, MA, USA).

### 4.3. Incubation of Cells with AuNPs and TiO_2_ NPs

Cells were incubated with anatase TiO_2_ NPs for 24 h at 20, 40, 60, 80, 160 μg/mL concentrations. All experiments were carried out at ambient light conditions. Cells were exposed to light only during NP incubation and for equal durations to minimize additional photoreactivity of TiO_2_ NPs. Additionally, ~1.6 × 10^15^ of AuNPs were added simultaneously to the incubation medium to make up 25% (*v/v*) of the 1 mL in each well of a 24-well plate for SERS analyses. For the WST-1 assay, the AuNP amount was approximately 0.16 × 10^15^ in the total 100 μL incubation medium of a 96-well plate.

### 4.4. WST-1 Assay

Following NP incubation for 24 h, cells were washed once with phosphate buffered saline (PBS) and incubated with WST-1 containing complete medium for 1 h at 37 °C, at 5% CO_2_ in a humidified incubator. After 1 h, supernatant was transferred to a new 96-well plate to prevent NP interference during absorbance measurement. Absorbance values were read by a microplate reader at 450 nm (ELx808, BioTek, Winooski, VT, USA). The % Cell viability of test groups were normalized to their respective non-exposed samples, setting the viability level of non-exposed samples to 100%. Two-tailed Student’s *t*-test for unpaired samples was applied to test the effect of NP incubation compared to control samples that were incubated with only AuNPs. Samples with a *p* ≤ 0.05 significance value were marked with a star sign.

### 4.5. SERS Experimental Setup

Upon 24 h of incubation, the NP incubated-cells that were seeded on CaF_2_ slides were rinsed with PBS and while the cells were still alive, the slides with the live cells were directly moved onto a polydimethylsiloxane (PDMS) coated polystyrene Petri dish for analysis. The PDMS coating suppressed the polystyrene peaks that could originate from the Petri dish. A drop of serum-free cell culture medium was added on CaF_2_ slides to prevent the cells from drying throughout SERS measurements. For all SERS measurements, a Renishaw inVia Reflex Raman spectrometer (Wotton under Edge, United Kingdom) equipped with a high speed encoded stage (Streamline™), a Leica DM2500 upright microscope and an 830 nm laser source with 1200 line/mm grating were used. The laser spot size was approximately 2.5 μm on the sample where Leica 20× long distance objective (N.A. = 0.40) was used. Because the sample was in liquid and a long distance objective was used, the laser power was kept at 150 mW with 2 s exposure time. Lower laser power did not generate any meaningful spectrum except noise, whereas the 150 mW high laser power did not damage the cells but gave high signal intensity. The measurements were carried out at a chosen area of approximately 30 × 30 μm with 2 μm step size. By the help of the high speed encoded stage, mapping an area of this size took about 4–5 min. The spectral acquisition range was set to 1473–478 cm^−1^ with a 0.9 cm^−1^ spectral resolution.

### 4.6. SERS Data Processing

A minimum of four cells were scanned on a CaF_2_ slide and a minimum of 50 cells were scanned for each test group. The spectra obtained from a single-cell were pre-processed and averaged to yield a spectrum representing that single cell. Acquired spectra were background corrected, smoothed by Savitzky-Golay filtering, vector normalized to unit 1 and cosmic spikes were removed by using the instrument software WiRE™ 4.2 (Wotton under Edge, Gloucestershire, UK). All the averaged spectra from each single cell of a test group were averaged once again to obtain a representative spectrum of that specific test group.

## Figures and Tables

**Table 1:** The list of the most prominent peaks in the SERS spectra and their assignments from the literature [37,48].

Peak (cm^−1^)	Peak Assignment
480	*Glycogen*
500–560	*S-S bond vibrations*
620	*C-C twist aromatic ring*
640	*C-S stretching and C-C twisting of proteins*
655	*C-S stretching mode of cystine* (*collagen type I*)
710	*Cholesterol, C-N* (*membrane phospholipid head*)
830	*Tyrosine*
853	*Proline*
885	*Tyrosine*
977	*C-C stretching* β*-sheet* (*proteins*)
1003	vs(*C-C*)*, symmetric ring breathing, Phenylalanine*
1018	*Glycogen*
1030	*Phenylalanine and changes in collagen*
1060	*Triglycerides, Lipids, Ceramide, Proline*
1130	*Phospholipid structural changes trans-gauche*
1204	*Hydroxyproline*
1216	*Amide III* (β*-sheet*)
1270	*Amide III,* α*-helix, collagen*
1317	*Twisting or bending mode of lipid/collagen*
1352	*Nucleic acid mode, Collagen content changes*
